# Ethnographic evaluation of usability, understandability, and acceptance of the MY PD-CARE digital tool to facilitate Parkinson’s disease symptom tracking and patients’ and care partners’ communications with the treating healthcare professional: the SELF-AWARE study

**DOI:** 10.1007/s10072-025-08342-0

**Published:** 2025-07-18

**Authors:** Angelo Antonini, Tove Henriksen, Amelia Hursey, Lars Bergmann, Juan Carlos Parra, Per Odin

**Affiliations:** 1 Neurodegenerative Disease Unit, Department of Neuroscience, Padua Neuroscience Center (PNC), University of Padova, Padova, Italy; 2 Movement Disorder Clinic, University Hospital of Bispebjerg, Copenhagen, Denmark; 3 Parkinson’s Europe, London, UK; 4 AbbVie Inc, North Chicago, IL USA; 5 Division of Neurology, Department of Clinical Sciences, Lund University, Skane University Hospital, Lund, Sweden

**Keywords:** Chronic, Digital health, Health communications, Mixed methods, Neurology, Neuroscience

## Abstract

**Background and aim:**

The MY PD-CARE digital tool is intended to empower people with Parkinson’s disease (PD) and care partners to actively identify and track changes in key symptoms of advancing PD and to facilitate discussions with healthcare professionals (HCPs). MY PD-CARE was adapted from MANAGE-PD, a validated, web-based tool that helps HCPs identify patients with inadequate symptom control. The SELF-AWARE (**S**tudy on **E**thnographic research and human factors eva**L**uation **F**or a tool to increase **AW**areness, self-**A**ssessment, and **R**eporting of PD pati**E**nts uncontrolled on oral medication) study investigated MY PD-CARE ease of use, understandability, and acceptance among people with PD and their care partners.

**Methods:**

SELF-AWARE was a non-interventional, cross-sectional, observational study. Participants (patients/care partners) assessed MY PD-CARE during 1-time virtual interviews conducted by trained medical anthropologists using qualitative ethnographic and human factor evaluation methods.

**Results:**

In 90.7% of interviews (43 patients; 31 care partners), ≥ 1 patient/care partner participant was comfortable with technology. Most participants understood the purpose/objective of MY PD-CARE and agreed it was simple and easy to use. Although the medical terminology was not fully self-explanatory to many participants, half found the glossary helpful. Approximately 60% indicated MY PD-CARE could have more value with free-text input. Participants agreed that MY PD-CARE is useful for tracking symptoms and encouraging discussions with HCPs.

**Conclusions:**

People with PD and their care partners perceived MY PD-CARE as useful and acceptable for tracking and increasing awareness of symptoms and facilitating discussions with HCPs. Participant feedback helped optimize the updated design of this digital tool.

**Supplementary Information:**

The online version contains supplementary material available at 10.1007/s10072-025-08342-0.

## Introduction

Parkinson’s disease (PD) is a chronic neurodegenerative disorder characterized primarily by motor dysfunction symptoms [1]. Despite the effectiveness of available symptomatic treatments, PD symptom progression can significantly impact the quality of life for those affected and their care partners [2–4]. The progression of PD varies widely, and certain motor complications can require individualized treatment [2, 5].

More people today want to actively participate in their healthcare decisions [6, 7]. They strive to enhance their self-management skills and collaborate with their healthcare professional (HCP) to make well-informed care decisions [6, 7]. Symptom tracking enables people to stay informed about their condition, detect progression signs, monitor treatment effectiveness, and communicate more effectively with their HCPs [8]. Digital technologies can help individuals with PD and their care partners identify symptoms and motivate them to discuss concerns with their HCPs [9, 10]. User-centric research is crucial for successful development of digital tools, helping ensure the final tool best meets the needs and preferences of the intended users, which can lead to improved adoption and more effective tool use [11].

Herein, we describe results from the SELF-AWARE (**S**tudy on **E**thnographic research and human factors eva**L**uation **F**or a tool to increase **AW**areness, self-**A**ssessment, and **R**eporting of PD pati**E**nts uncontrolled on oral medication) study, a non-interventional, observational, user-centric research study in which people with PD and their care partners evaluated the usability, understandability, acceptance, and perceived usefulness of a digital prototype of MY PD-CARE—a novel tool developed to help people with PD and their care partners track PD symptoms and facilitate discussions about their findings with their HCPs. MY PD-CARE was developed to be an abbreviated “patient version” of the MANAGE-PD tool, which is a validated, web-based clinical tool that helps HCPs identify people with PD who have symptoms not adequately controlled by current oral medication and who may be eligible for device-aided therapies [12–14]. SELF-AWARE study findings will help optimize future iterations of the MY PD-CARE tool [15] that are convenient (accessible at any time), user friendly, and valuable for people with PD and their care partners. It is important to highlight that MY PD-CARE is not intended to diagnose PD or provide treatment recommendations—it is designed to record and summarize patient responses, which can then be shared or discussed with HCPs.

## Methods

### Study design and participants

**Fig. 1:**
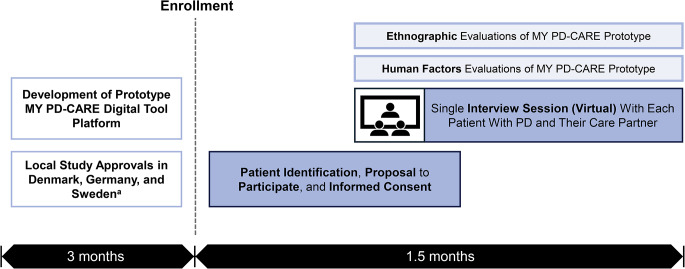
SELF-AWARE study flow. SELF-AWARE (**S**tudy on **E**thnographic research and human factors eva**L**uation **F**or a tool to increase **Aw**areness, self-**A**ssessment, and **R**eporting of PD pati**E**nts uncontrolled on oral medication). ^a^Spain was initially included as a prospective country for recruitment but was later excluded due to the complexities of the local regulatory environment

SELF-AWARE was a cross-sectional study conducted in Denmark, Germany, and Sweden (Fig. 1). The study used a mixed-methods approach through combined ethnography and human factors survey methodologies to validate the MY PD-CARE digital tool prototype (content and platform).

Study participants were recruited between December 13, 2021, and March 4, 2022, via patient organization outreach (primary method) and supplemental recruiting efforts, including newspaper and Facebook advertisements. People who were diagnosed with PD at least 3 years before the study and who had a primary care partner willing to participate engaged in a 1-time native-language virtual interview. Interviewers were provided with a discussion guide to help guide the conversation (Supplementary Table 1). Patients enrolled in a concurrent clinical trial or who were previously treated with any deviceaided therapy were not eligible to participate.

This study was conducted in accordance with the ethical principles originating in the European Medicines Agency Module XVI Guideline on Good Pharmacovigilance Practices. The study was approved by an independent ethics committee as required by local laws and regulations. All study participants provided written informed consent.

### Assessments

Each interview was conducted by 1 or 2 trained medical anthropologists. The interviews were approximately 1 h in duration and were conducted virtually with patients participating from their own homes. Interviewers were provided with a thematic discussion guide to structure and organize the conversation (Supplementary Table 1). The discussion guide allowed the interviewer to accommodate the normal flow of a conversation while ensuring that all themes and topics are discussed through simple, open-ended questions. This structure allowed participants to respond in their own words to reflect on their unique experiences.

During the initial part of the interview, interviewers collected information on patient demographics, medical history, care routines, and comfort level with technology.

During the next part of the interview, participants evaluated the digital MY PD-CARE tool prototype questions and platform (Supplementary Fig. 1) via desktop computer, tablet, or mobile device. Qualitative ethnographic evaluations (primary study objective) included assessments of (1) understandability of the tool objective, tool questions, additional information and definitions of medical terms, and the printable final summary report; and (2) acceptance and perceived usefulness of the digital tool overall. Quantitative human factor evaluations (secondary study objective) included assessments of (1) understanding tool functionality; (2) general usability of the tool, its navigation, and self-explanatory nature; and (3) management of the summary output.

### Data management

Idea Couture, Inc (a Cognizant company) managed study data, provided de-identified data to the study sponsor (AbbVie), and retained video recordings and survey responses for as long as reasonably necessary to complete the project in accordance with applicable laws and rules on statute of limitations. All saved files used de-identified participant IDs. Idea Couture, Inc verified the data to ensure accuracy. Interviews were conducted virtually on the Cisco WebEx platform that used end-to-end encryption.

### Statistical analysis

Approximately 13 patient/care partner dyads in each of the 3 participating countries (Denmark, Germany, and Sweden) were planned for recruitment. The goal of this qualitative ethnographic research was to discover directional accuracy, not statistical reliability. Therefore, the data collected reflect the depth of experience rather than the breadth of participants. Recognizing this study includes a specific population (i.e., people living with PD) for research within a fixed timeline, the study sample size represents what was considered to be a minimum number of participants needed to glean in-depth insights. Consistent with this study size rationale, no formal statistical testing was done in this study, and data are summarized using descriptive statistics.

## Results

### Participants

#### Interviews

Native-language medical anthropologists conducted 43 interviews in Denmark (*n* = 20), Germany (*n* = 19), and Sweden (*n* = 4) between January and March of 2022. Of the 43 interviews, 31 (72.1%) included the patient with PD and their care partner (i.e., *n* = 31 patient/care partner pairs), while the remaining 12 interviews (27.9%) were conducted with the patient alone. Participants most frequently used the desktop computer format (86.0% [*n* = 37]) followed by tablet and mobile formats (7.0% [*n* = 3] each).

#### Demographic characteristics

Most of the 43 patient participants were aged between 50 and 70 years (65.1%), were male (60.5%), and/or had been diagnosed with PD at least 6 years before enrolling in this study (67.4%) (Table 1). Of the patients providing information on their education and/or work status, 68.2% (15/22) reported completion of trade/specialist (college) training or achievement of a university or graduate school degree, and 80.0% (28/35 with existing data) reported being retired. The majority of the 31 care partners interviewed were female (67.7%), and nearly all (96.8%) were the patient’s spouse or partner.

**Table 1:** Patient and care partner demographic characteristics

Characteristic	
Patients	(*N* = 43)
Age, years, mean (SD)	67 (6.3)^a^
Age group, n (%)	
≤ 65 years > 65 years 50–70 years 70–90 years	12 (46.2)^a^ 14 (53.8)^a^ 28 (65.1) 15 (34.9)
Sex, n (%)	
Male Female	26 (60.5) 17 (39.5)
Time since PD diagnosis, n (%)	
3–5 years 6–8 years 9 + years	14 (32.6) 18 (41.9) 11 (25.6)
Education,^b^ n (%)	
Some high school Completed high school Some college Completed college University degree Graduate degree	4 (18.2) 1 (4.5) 2 (9.1) 9 (40.9) 4 (18.2) 2 (9.1)
Employment,^c^ n (%)	
Full time Part time Retired	3 (8.6) 4 (11.4) 28 (80.0)
Comorbidities,^d^ n (%)	
Blood pressure abnormalities Heart issues Diabetes Incontinence Hypothyroidism Heartburn Cancer Depression Sleep issues Hypothyroidism	6 (33.3)^e^ 3 (30.0)^f^ 2 (10.0)^g^ 1 (5.0)^g^ 1 (5.0)^g^ 1 (5.0)^g^ 1 (5.0)^g^ 1 (5.0)^g^ 1 (5.0)^g^ 1 (5.0)^g^

Care Partners	(*N* = 31)
Sex, n (%)	
Male Female	10 (32.3) 21 (67.7)
Patient’s spouse/partner, n (%)	30 (96.8)
Patient’s parent, n (%)	1 (3.2)

PD, Parkinson’s disease

^a^*n*=26 (specific age unknown for remaining patients)

^b^*n*=22

^c^*n*=35

^d^Patients may have reported no comorbidity or more than 1 comorbidity

^e^*n*=18

^f^*n*=10

^g^*n*=20

#### Patient degree of independence

Of the 43 patients, 34 (79.1%) required help with day-to-day activities. The majority of patients reported they needed help with most (32.6%) or a few (41.9%) day-to-day activities, with some (2.3%) needing help for all activities.

#### Current PD management

Neurologist appointments varied in frequency from every 3–18 months, with appointment lengths ranging from 5 to 60 min. During all 43 interviews, patients and care partners expressed there was significant pressure for appointments to be effective in managing PD. They also indicated a desire for more time with the neurologist:

*“There is not a lot of dialogue [at the appointment], you are a number in the line. You come in, and she asks how you have been, and you might say, ‘I feel kind of the same as the last time; I might have experienced a little more shaking’…‘well, that is fine, then we will just increase the dose a bit,’ and then that is that.”*

*“I would absolutely wish to see changes. Seventy-four is not an age where you say life’s over, yet I wish I could get more from the doctor, but I’m not sure how to find that.”*

Despite the limited time patients and care partners reported having with their neurologist, there were wide variations in the level of preparation they made before their appointment. Only 11.6% of participants (*n* = 5) kept detailed records to document the times, medications, number of doses, and symptoms of their PD, 20.9% (*n* = 9) spent only a limited amount of time preparing for the HCP interaction (i.e., making notes and writing down questions), and 41.9% (*n* = 18) spent no time preparing and relied on their memory during HCP interactions. The remaining 25.6% of participants (*n* = 11) did not comment on their preparation.

Approximately half the participants (53.5% [*n* = 23]) reported they played an active role in working with their HCP to develop a PD care plan. The remaining participants (46.5% [*n* = 20]) indicated they did not but would like to play an active role. There was no substantial difference across the 3 countries regarding HCP management of PD.

#### Technology (digital) proficiency

**Fig. 2:**
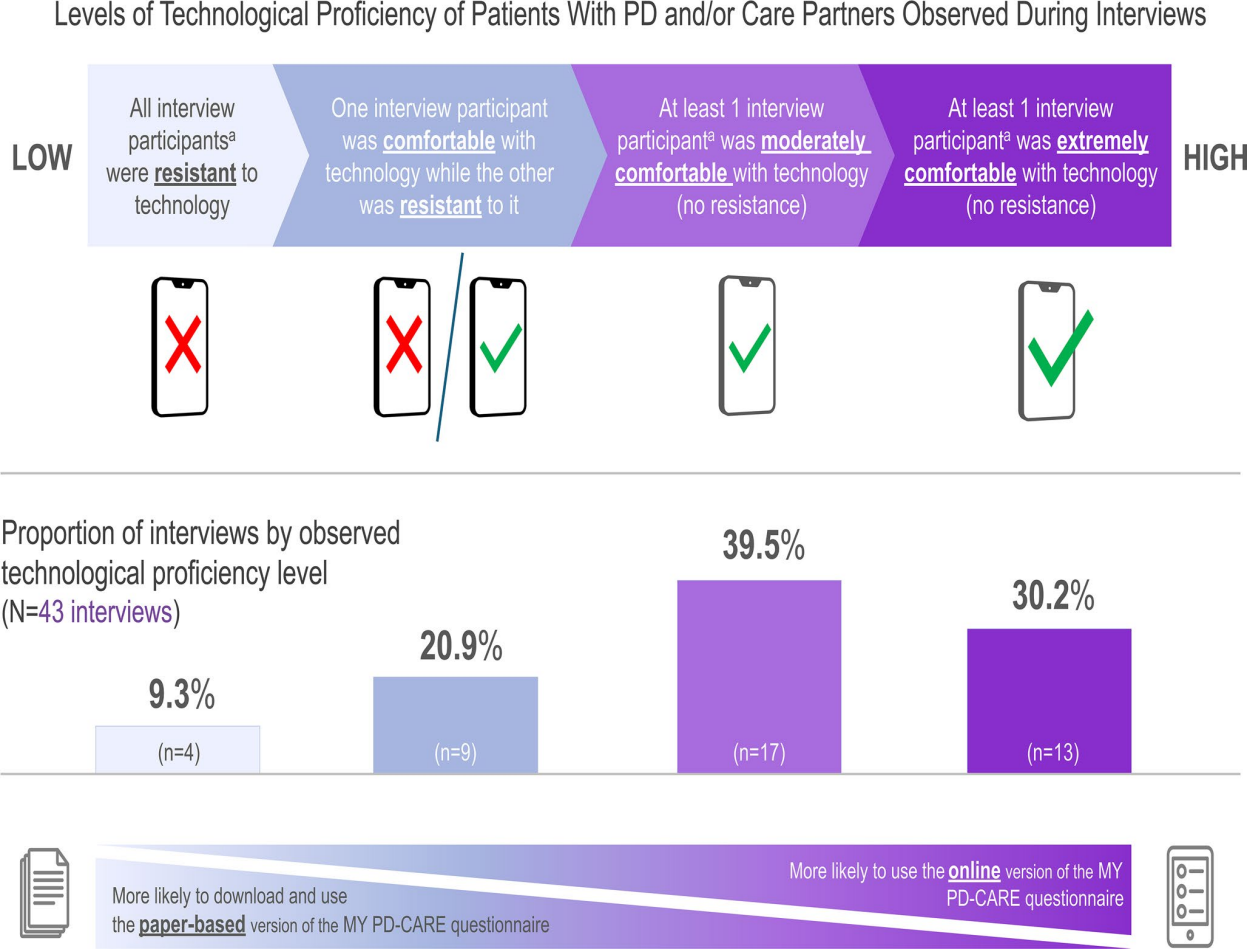
Technological (digital) proficiency of patients with PD and/or their care partners. PD, Parkinson’s disease. ^a^Refers to interviews conducted with patient/care partner pairs (*n* = 31 interviews total) and/or interviews conducted with the patient alone (*n* = 12 interviews total)

As patients and their care partners are not necessarily equally technologically proficient, patient and (if available) care partner participants in all 43 interviews were evaluated for technological proficiency (Fig. 2). In 90.7% of interviews (*n* = 39), ≥ 1 participant (patient or care partner) was comfortable using digital technology across multiple devices. In 9.3% of the interviews (*n* = 4), the patient and care partner were both resistant to digital technology and actively avoided it in daily life. Study participants who were resistant to digital technology tended to prefer the paper-based questionnaire of MY PD-CARE. Factors that affected technological proficiency included disease severity, previous careers with the absence or presence of technological requirements, and socioeconomic status.

### Qualitative ethnographic assessments

#### General usability

**Fig. 3:**
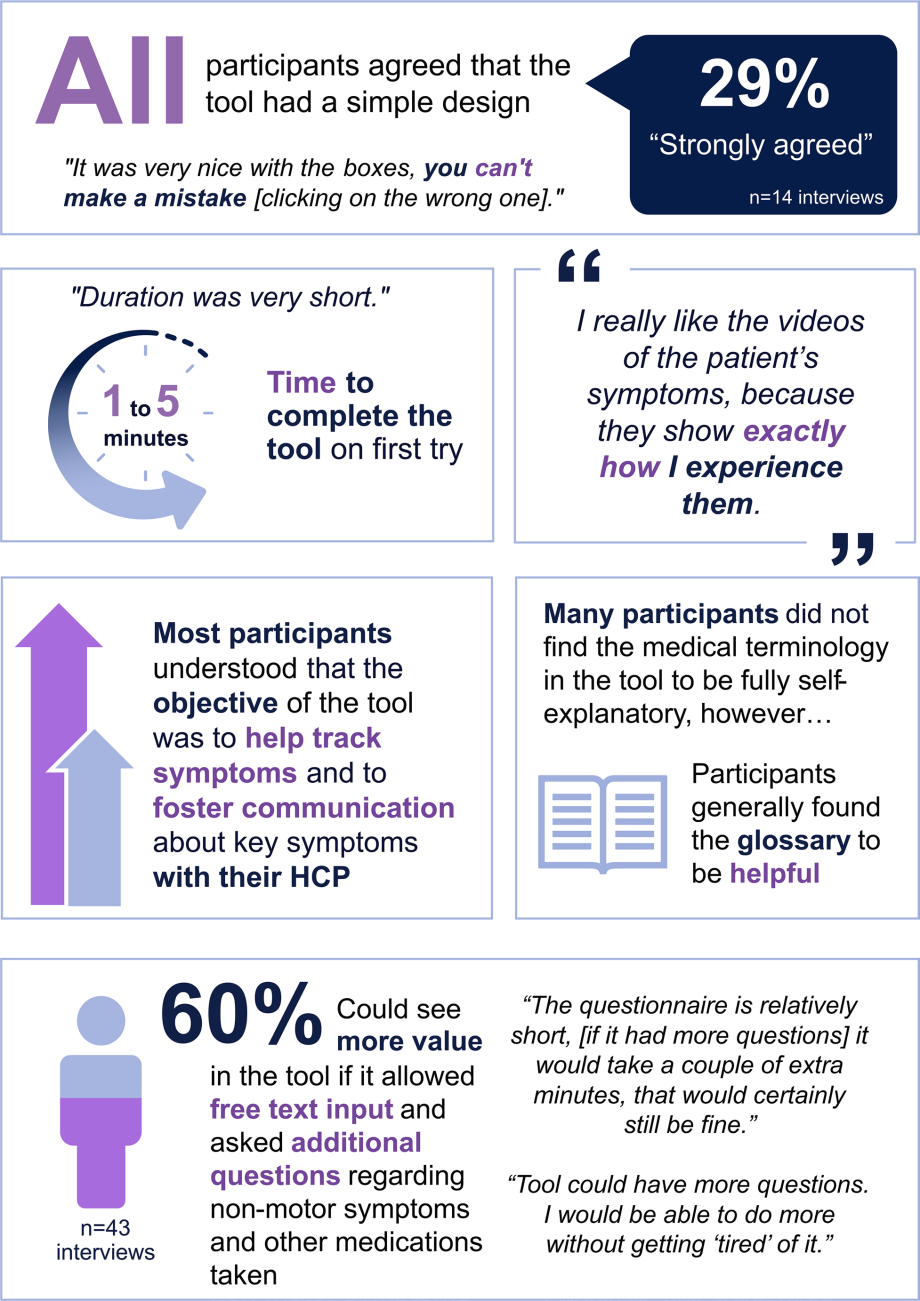
Patient and care partner evaluation of the MY PD-CARE digital tool prototype: ease of use and understandability. HCP, healthcare professional

In general, patient and care partner participants perceived the digital prototype tool (Supplementary Fig. 1) to be easy to use with a simple design that provided the right amount of information per page (Fig. 3). All participants were able to complete the questionnaire within 5 min on their first try.

#### Understandability

There were 2 qualitative themes that participants reported regarding the tool’s objective (Fig. 3). The first was to increase patient and care partner awareness of current and future PD symptoms (the latter was a common theme for patients with relatively mild PD):

*“I like the idea behind the [MY PD-CARE] tool and understand that in the longer run, when symptoms worsen, it would make sense to track like this [with MY PD-CARE].”*

*“[The MY PD-CARE tool] would be extremely helpful in tracking my symptom management because it increases engagement with my symptoms and makes you relive and reflect on specific moments.”*

*“I think it’s very important that I can evaluate myself independently from the doctor/expert.”*

The second theme that participants reported regarding the tool’s objective was sharing and discussing collected symptom data with their neurologist to foster informed communication and shared decision-making. All patients wanted to track their symptoms and prepare for their appointments with their neurologist but were unsure how to do so. Participants from 14.0% of the interviews (*n* = 6) shared unsolicited feedback indicating that they understood the tool’s purpose was to inform the neurologist about whether a treatment change was necessary based on their symptoms:

*“With MY PD-CARE, we are better able to communicate with the neurologist, and I think that’s the purpose: we’re talking in the same way.”*

*“[MY PD-CARE] can only be positive. The more you know, the more you discuss, the more you can achieve.”*

*“MY PD-CARE might help me bring more to the table during the sessions with my neurologist.”*

#### Perceived acceptance and usefulness

Many participants agreed that the MY PD-CARE prototype (Supplementary Fig. 1) would be a useful tool for tracking symptoms and encouraging discussion with their neurologists. However, others indicated that the questionnaire was too limited in its functionality and/or scope, with participants from 26 interviews (60.5%) surmising that if the tool’s objective was to track symptoms and improve communication with neurologists, the utility of the tool may be improved by introducing some flexibility and asking additional questions or allowing text input (Fig. 3):

*“People get different PD medication, but that’s missing in the tool. I’d expect it to ask ‘which medications do you take?’ rather than something specific like levodopa only.”*

*“I am wondering, so, PD symptoms are made up of 2 parts. One part is the physical, and the other is the cognitive. The cognitive has a big impact on a lot of PD patients, and the tool did not have very much about this.”*

Importantly, participants were generally open to adding more questions as needed and did not believe it would discourage them from using the tool. Many participants were also amenable to using the tool in a way that was most useful to their treatment regimen and neurologist. The most common case was when medications or symptoms were changing. Patients and care partners predicted they would use the tool daily, weekly, or monthly (if it contained more questions beyond motor symptoms) leading up to neurologist appointments.

### Quantitative human factor research assessments

#### Understandability of MY PD-CARE tool questions and medical terminology

Many participants had difficulty understanding the terminology used in the prototype tool’s questions (e.g., “dyskinesia,” “motor fluctuations,” “Off-time”) (Supplementary Fig. 1; Fig. 3). Participants from almost half the interviews (44.2% [*n* = 19]) stated that the medical terminology used could be intimidating and suboptimal for older adults. In contrast, participants from one-third of the interviews (30.2% [*n* = 13]) indicated that using the terminology from MY PD-CARE questions could help them better communicate with their neurologist. Other participants (20.9% of interviews [*n* = 9]) were familiar with the medical terminology due to their medical background before retiring (e.g., former dentist, neurologist) and through independent research of PD. Overall, participants from 79.1% of interviews (*n* = 34) did not use medical terminology in their day-to-day vocabulary to describe their PD symptoms, and some had difficulty pronouncing the words.

The glossary, located to the right of each symptom question, was designed to help users understand the medical terminology used (Supplementary Fig. 1). Participants found the glossary either on their own or were directed to it by a researcher. Once they accessed it, participants who believed that the descriptions clearly defined the medical terms—about half of the study participants found the glossary “intuitive”—expressed appreciation for this useful feature of the MY PD-CARE tool:

*“I have a general idea of what it [the medical terms] means, but when it’s explained again there, that’s great.”*

*“The extra texts to describe the medical terms was helpful.*”

However, some participants found the glossary difficult to see due to the color design (light grey backdrop) and/or the small font size used:

*“The glossary is too small and difficult to read*,* especially for [Patient X].”*

*“[Patient Y] suggested that it might be good to make it a bit more visual and easy to see.”*

Many participants showed some level of medical terminology misinterpretation, which typically occurred when patients had not yet seen the glossary. The researchers noted that the most frequent misinterpretations were related to confusion about the meanings of motor fluctuations and dyskinesia.

Interviewers aimed to determine whether including videos that explain the symptoms assessed in the MY PD-CARE questionnaire could enhance users’ understanding of the tool in future iterations. Draft videos, shared with study participants separate from the MY PD-CARE tool during the interview, were indeed found to be helpful by some patients; for example, 1 patient stated:

*“I really like the videos of the patient´s symptoms because they show exactly how I experience them.”*

#### Understanding of the MY PD-CARE tool functionality

All study participants found the MY PD-CARE tool easy and clear to navigate. Users could use the “back” button to revisit previous pages to correct or review previous content, and they appreciated the size of the response buttons:

*“[Care partner X] likes the size of the button, ‘They are so big that you won’t miss them when you try to click.”*

*“It was easy to read everything except for the glossary; it was too small.”*

*“It was very nice with the boxes; you can’t make a mistake [click the wrong one].”*

#### MY PD-CARE summary report

**Fig. 4:**
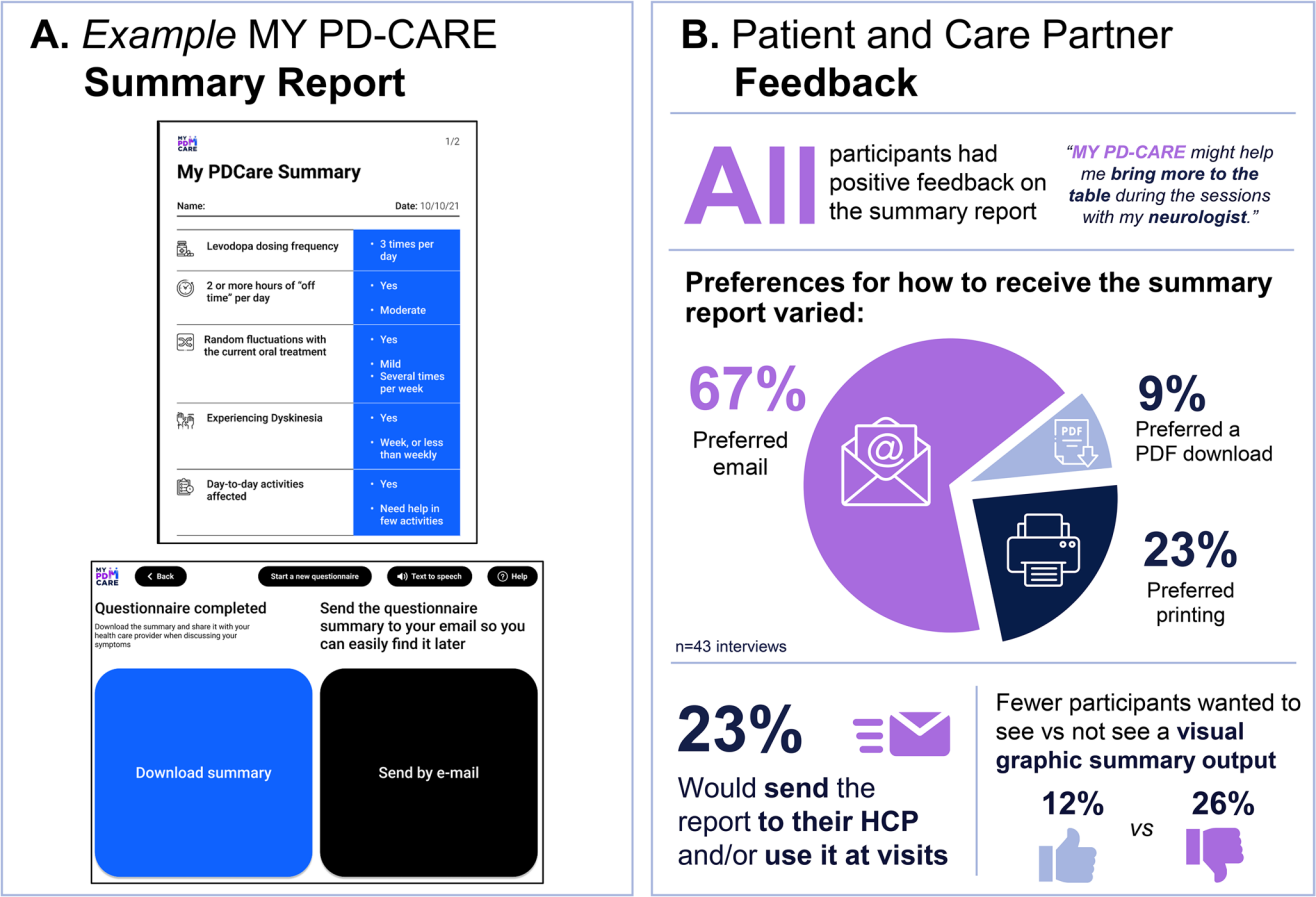
Patient and care partner evaluation of the MY PD-CARE digital tool prototype: summary report. HCP, healthcare professional

Patients and care partners provided positive feedback on the availability of options to receive, store, or share the summary report (Fig. 4). When given the choice to either email or download the summary output after completing the questionnaire, most participants preferred email (67.4% [*n* = 29]). Other participants (9.3% [*n* = 4]) expressed a preference for downloading the summary, as they preferred to keep all their PD-related documents organized in folders. Almost one-quarter of participants (23.3% [*n* = 10]) expressed a desire to print the summary and bring it to their neurologist. Some participants (14.0% [*n* = 6]) did not find the reminder feature helpful.

All participants had positive feedback on the summary report. Some participants (23.3% [*n* = 10]) expressed a desire to send the summary to their neurologist and/or keep it for their records and present it at future appointments. Others (11.6% [*n* = 5]) expressed interest in viewing their progress over time in a more visual, graphic representation. However, some participants (25.6% [*n* = 11]) specifically stated that they would not consider a visual representation of their results productive; given PD is a degenerative disease, they would not like to see their decline in this visual manner.

Participants were also shown an alternative layout of the tool with a different visual configuration, displaying the question on the left and answer buttons on the right (as opposed to the current layout with the question at the top-middle and answer buttons at the bottom-middle). There was no substantive difference in patient or care partner preference for either layout.

## Discussion

The SELF-AWARE study assessed the usability, understandability, acceptance, and perceived usefulness of a digital prototype of MY PD-CARE—a novel tool designed to empower people with PD and their care partners to have a more active role in disease management. Using user-centric research survey methods, this study showed that the prototype tool was generally well received.

The primary objective of the SELF-AWARE study was to evaluate the content usability and digital fluency of the MY PD-CARE tool’s questions and formats, and the usefulness of the tool for patients and care partners using ethnographic survey methodologies. Most participants agreed that the tool had a simple design, was easy to navigate, and was understandable due to the simple design interface, taking ≤ 5 min to complete. The general objective of the tool (i.e., to track/increase awareness of key symptoms and improve communication with their neurologist) was clear to most participants. Because MY PD-CARE was designed as a stand-alone tool for independent use by patients and care partners, user understandability of the tool could be enhanced in subsequent iteration(s) by clearly communicating the tool’s objectives on a starting screen so that users would consistently and fully understand the tool’s objective.

The secondary objective of this study was to evaluate the platform usability, understandability, and handling of the MY PD-CARE tool via human factor methodologies. While some participants found the medical terminology in the tool unclear, they valued the accompanying glossary, which helped them use correct terminology with their neurologist. None of the participants reported that the tool was difficult to use or navigate. The summary report was well received; some participants wanted to email or download the summary output for printing or filing with other PD documents. Other participants believed the printed report would allow them to view their progress over time. The results and the recommendations (presented below) for tool improvements emphasize the importance of user-centric research in healthcare tool development. This type of research ensures that the final tool meets the needs and preferences of the intended users, supporting successful adoption.

The range of recommendations from participants for the next iteration of MY PD-CARE highlights the diverse nature of PD and the varying levels of knowledge about the condition among patients and care partners. Some study participants suggested that expanding the MY PD-CARE tool to capture additional disease information (e.g., non-motor symptoms, medication use, HCP questions) would improve its effectiveness. However, expanding the questionnaire to include evaluation of other symptoms would modify the intended objective of the tool (i.e., to monitor key symptoms indicating the disease is uncontrolled on oral PD medication) and result in a misalignment of questions used to screen for inadequate symptom control in the MY PD-CARE tool vs. the validated MANAGE-PD HCP tool [12] (from which the MY PD-CARE tool was derived). Adding additional questions could be perceived as burdensome and negatively impact its adoption. Conversely, the recommendation for an open-text option could preserve the intended objective of the tool while providing patients and care partners with the opportunity to capture additional information.

Patients and care partners also recommended that future iterations of the MY PD-CARE tool use clearer language to improve understanding of medical terminology. Motor fluctuation terminology was often misinterpreted and caused confusion, highlighting the need for education. Describing the key symptoms via a glossary in a more prominent manner before administering the questionnaire may reduce the time needed to answer the questions and provide higher-quality responses. As shown in Supplementary Fig. 2, participant suggestions were considered, with several important enhancements implemented in the EU MY PD-CARE digital tool released in early 2023 [15]. Notable enhancements included the addition of a clear and visually engaging description of the tool’s objectives on an early screen (in both text and video options) so that users could better understand the tool’s objective (Supplementary Fig. 2A), an improved screen design and flow to help users more easily understand (with both text and video descriptions) the medical terminology (Supplementary Fig. 2B), and the addition of an open-text option, the contents of which are included in tool’s summary report (Supplementary Fig. 2C).

Care partners were helpful in collecting accurate and objective PD information from patients. Often, care partners led navigation of the tool—reading questions and inputting answers—while in other cases, patients and care partners collaborated equally. This partnership helped clarify ambiguous symptoms, resulting in responses that were often “somewhere in the middle” rather than solely from the patient’s or care partner’s perspective. The most important takeaway from this insight was that the care partner’s involvement likely facilitated more objective responses than would have been given if the patient had been interviewed alone. Interestingly, patients in advanced stages recommended co-adoption of the MY PD-CARE tool, with some stating they would only use it with their care partner.

A key strength of this study was the mixed-methods approach used (utilizing combined ethnographic and human factor survey methodologies) to optimize patient and care partner adoption of the MY PD-CARE tool. Other important strengths were that all research was performed by trained medical anthropologists and that all interviews were conducted in participants’ native language.

There were several study limitations. Information on disease severity and cognitive function was not collected. Additionally, collection of qualitative data through virtual interviews was necessitated by the COVID-19 pandemic, possibly introducing bias toward participants with higher technological proficiency than the general PD population. While the in-depth interviews were intended to provide deep insights, the lack of quantification of data and the limited number of included participants and countries restrict the generalizability of the findings. Patients with cognitive problems may have been unable to understand the significance of specific questions asked by the interviewer, underscoring the importance of care partner involvement in symptom assessment and tracking. Finally, the paper-based (downloadable) version of the MY PD-CARE prototype was not evaluated in this study, though the concept of having this option was well received by participants.

## Conclusions

The prototype of the MY PD-CARE digital tool received positive feedback from people with PD and care partners, particularly in terms of its ease of use, understandability, acceptance, and perceived usefulness for symptom tracking. Study participants also believed that using the MY PD-CARE tool could increase symptom awareness and help them discuss their symptoms with their neurologist. Participant feedback led to several enhancements in the EU MY PD-CARE tool, and ongoing user input will be integral for further optimization. This study highlights the importance of including people with PD and their care partners in the design process of a future digital tool to help ensure user needs will be met.

## Electronic supplementary material

Below is the link to the electronic supplementary material.

Supplementary Material 1

## Data Availability

AbbVie is committed to responsible data sharing regarding the studies we sponsor. This includes access to anonymized individual and study-level data (analysis data sets), as well as other information (e.g., protocols, clinical study reports, or analysis plans), as long as the study is not part of an ongoing or planned regulatory submission. This includes requests for study data for unlicensed products and indications. These study data can be requested by any qualified researchers who engage in rigorous, independent, scientific research, and will be provided following review and approval of a research proposal, Statistical Analysis Plan (SAP), and execution of a Data Sharing Agreement (DSA). Data requests can be submitted at any time after approval in the United States and Europe and after acceptance of this manuscript for publication. The data will be accessible for 12 months, with possible extensions considered. For more information on the process or to submit a request, visit the following link: https://vivli.org/ourmember/abbvie/, then select “Home.”
