# Supplementary material for: Ethnographic evaluation of usability, understandability, and acceptance of the MY PD-CARE digital tool to facilitate Parkinson’s disease symptom tracking and patients’ and care partners’ communications with the treating healthcare professional: the SELF-AWARE study
Source: Neurol Sci. 2025 Jul 18;46(10):5061–71. doi: 10.1007/s10072-025-08342-0 (PMC12488775; doi:10.1007/s10072-025-08342-0)
Supplement: Supplementary file 1 — Supplementary Material 1 [file 10072_2025_8342_MOESM1_ESM.pdf]

SUPPLEMENTARY MATERIALS

Ethnographic evaluation of usability, understandability, and acceptance of the MY PD-CARE digital tool to facilitate Parkinson's disease symptom tracking and patients' and care partners' communications with the treating healthcare professional: The SELF-AWARE study

Angelo Antonini[1] • Tove Henriksen[2] • Amelia Hursey[3] • Lars Bergmann[4] • Juan Carlos Parra[4] • Per Odin[5]

[1]Neurodegenerative Disease Unit, Department of Neuroscience, Padua Neuroscience Center (PNC), University of Padova, Padova, Italy

[2]Movement Disorder Clinic, University Hospital of Bispebjerg, Copenhagen, Demark

[3]Parkinson's Europe, London, UK

[4]AbbVie Inc., North Chicago, IL, USA

[5]Division of Neurology, Department of Clinical Sciences, Lund University, Skane University Hospital, Lund, Sweden

Corresponding author:

Angelo Antonini, Neurodegenerative Disease Unit, Department of Neuroscience, Padua Neuroscience Center (PNC), University of Padova, Padova, Italy.

Email: angelo.antonini@unipd.it

Supplementary Table 1. SELF-AWARE study interview discussion guide.

Part 1A: <u>Life With Parkinson's Disease</u> (≈5 minutes)

Objective: To understand the context and environment of an individual living with Parkinson's disease and that of their care partner

Q1. Could you tell me a bit about your journey of living with Parkinson's disease?

*[**Interviewer**]*

- *Probe on experiences around diagnosis and treatment journey, with a focus on the emotional aspects from a patient and care partner perspective*

Q2. How have you managed your Parkinson's disease?

*[**Interviewer**]*

- *Probe on how their expectations, day-to-day challenges, and approaches to treatment have evolved over time, as well as aspects of management that have created the biggest challenges*
- *Probe on the impact from a care partner perspective*

Part 1B: <u>Medical Interactions</u> (≈5 minutes)

Objective: To understand the individuals' experiences with healthcare system, HCPs, and approaches to disease management

Q1. How is your relationship with your HCPs? Neurologist, gastroenterologist, etc?

a. How do conversations typically go? How comfortable are you (ie, are you nervous, forget to explain or comment on anything? Do you find them useful? Do you prepare for the appointment? If so, how?
b. How often are your appointments? How long are your appointments?
c. What role do they play in your Parkinson's disease care and management?

Q2. What role does your physician play in supporting, advising, and helping you when it comes to managing symptoms?

Q3. How could your appointments with your HCP be improved?

Q4. What does current PD management look like?

*[**Interviewer**]*

- *Probe on level of preparation on patient's part for interactions with HCPs*
- *Ask for a list of tools, tips, or other techniques they currently use for benchmarking (any materials that have been shared by HCP; benefits and challenges to them)*
- *Probe on what types of technology (if any) they use to manage and monitor symptoms*

Tool Introduction

Objective: To Introduce the tool to the patient and care partner and allow them to explore the tool by completing an assessment

[Interviewer]

- *Introduce the tool to patient and care partner via screen share*
- *Record how long the assessment takes from the first tool question to the last (in minutes):* ___
- *Create a numbered list of questions (if any) that patient and care partner had while completing the assessment:* ___
- *Capture any spontaneous comments or reactions from the patient and care partner as they move through the tool*

Part 2: Tool Clarity and Communication

Objective: To understand how patients and care partners interact with the tool and their level of understanding

Q1. What are your first impressions of the tool?

[Interviewer]

- *Probe for their level of understanding, ability to answer questions, perspective*
- *Probe on thoughts about assessment length in terms of time, number of questions*

Q2. Looking at this tool, is there anything that helped/hindered your understanding?

[Interviewer]

- *Walk through the tool with the patient and care partner and probe on the understandability of the tool*
- *Ask them to flag any questions that resonated well and/or created a pain point for them*
- *Ask if the question wording was clear and easy to understand*

Q3. Did you understand the medical terms and definitions? Were any unclear?

[Interviewer]

- *List any medical terms that patients and care partners reported were unclear and why*

Q4. How well do you understand the medical terms and definitions provided?

[Interviewer]

- *Assess whether patients and care partners have a low (poor), moderate (some), or high (clear) understanding of medical terminology and symptom descriptions*

Q5. What are your impressions of the final output/summary?

[Interviewer]

- *Does the patient and care partner understand the purpose of the final output/summary*
- *Probe on how they would manage output. Would they print/save/copy etc?*
- *Did the patient indicate a preference for the paper questionnaire over the digital tool*
  - *Does their current physical functioning impact their preference?*

Q6. Overall, how did you find the experience of using this tool?

[Interviewer]

- *Rate the patient and care provider on how much support they required during their interactions with the tool in terms of understandability:*
  - *Overall comprehension was LOW: Patient and care partner asked a lot of questions and struggled in completing the self-assessment*

- *Overall comprehension was <u>MODERATE</u>: Patient and care partner asked several questions*
- *Overall comprehension was <u>HIGH</u>: Patient and care partner clearly understood the tool and asked few questions*

– *If comprehension differed between patient and care partner, explain nature of discrepancies: _____*
– *Comments: _____*

Part 3: <u>Usability</u>

Objective: To uncover any pain points and benefits the tool has for patients and care partners, and determine what steps can be taken to overcome them

*[**Interviewer**]*
– *Explain to the patient and care partner that you will be reading several statements about the usability and user-friendliness of the tool*
– *Explain the rating scale of 1 (Strongly Disagree) to 5 (Strongly Agree)*

Indicate your level of agreement with the following statement on a scale of 1 (Strongly Disagree) to 5 (Strongly Agree)

1. It is easy to learn how to use this tool

<u>Strongly Disagree</u>	*<u>Disagree</u>*	*<u>Neutral</u>*	*<u>Agree</u>*	*<u>Strongly Agree</u>*
1	*2*	*3*	*4*	*5*

2. The visual design is simple

<u>Strongly Disagree</u>	*<u>Disagree</u>*	*<u>Neutral</u>*	*<u>Agree</u>*	*<u>Strongly Agree</u>*
1	*2*	*3*	*4*	*5*

3. The functions were self-explanatory and intuitive (eg, definition pop-outs, buttons)

<u>Strongly Disagree</u>	*<u>Disagree</u>*	*<u>Neutral</u>*	*<u>Agree</u>*	*<u>Strongly Agree</u>*
1	*2*	*3*	*4*	*5*

4. The tool is well organized

<u>Strongly Disagree</u>	*<u>Disagree</u>*	*<u>Neutral</u>*	*<u>Agree</u>*	*<u>Strongly Agree</u>*
1	*2*	*3*	*4*	*5*

5. Navigating through the tool was easy

<u>Strongly Disagree</u>	*<u>Disagree</u>*	*<u>Neutral</u>*	*<u>Agree</u>*	*<u>Strongly Agree</u>*
1	*2*	*3*	*4*	*5*

6. The feature to explain medical terms was intuitive

Strongly Disagree	*Disagree*	*Neutral*	*Agree*	*Strongly Agree*
1	*2*	*3*	*4*	*5*

Q1. Is there anything that makes the tool user friendly? Difficult to use?

*[**Interviewer**]*

- *If necessary, walk through the tool with the patient and care partner again*
- *Probe on tool usability*
- *Ask them to flag any components that resonated well and/or created a pain point from their perspective*

*[**Interviewer**]*

- *Rate the patient and care partner on how much support they required during their interactions with the tool in terms of usability:*
  - *Overall usability was LOW: Patient and care partner required a lot of support, struggled to complete the self-assessment, and made many errors*
  - *Overall usability was MODERATE: Patient and care partner asked several questions and made a few errors but were able to recover*
  - *Overall usability was HIGH: Patient and care partner required very little support and completed the self-assessment efficiently*
- *If usability differed between patient and care partner, explain nature of discrepancies:* _____
- *Comments:* _____

Part 4: Acceptance and Perceived Usefulness

Objective: To gain insight into the usefulness of the tool, future iterations for the tool, and positioning of the tool in their disease management

Q1. Do you feel you have the capacity to *accurately* answer each of the questions?

a. No

Explain: _____

b. Yes

Explain: _____

Q2. How does this tool compare to the current approaches you use to track your symptoms?

*[**Interviewer**]*

- *Probe on where this tool stands in comparison to other techniques that they use to monitor symptoms*

Q3. If available, would you incorporate this self-assessment tool into your disease management?

a. No

Explain: _____

b. Yes

Explain: _____

[Interviewer]

- ***For those that answered <u>yes</u>***, *probe on how they could see themselves incorporating this tool into their disease management if it were available, and ask additional questions:*
  - How often would they use it? (eg. monthly, daily for week leading up to appointments, etc.?
  - In what environment would they use this tool? (eg, at home, at doctor's office, etc.)?

Q4. Knowing yourself, how useful would this tool be in tracking your symptom management?

a. Extremely unhelpful
b. Somewhat unhelpful
c. Neutral
d. Somewhat helpful
e. Extremely helpful

[Interviewer]

- *Ask why they chose their answer*
- *Ask patient and care partner to expand on barriers and/or benefits*

Q5. How useful would this be in your treatment if you came to your doctor's appointments prepared with this tool?

a. Extremely unhelpful
b. Somewhat unhelpful
c. Neutral
d. Somewhat helpful
e. Extremely helpful

[Interviewer]

- *Ask why they chose their answer*
- *Ask patient and care partner to expand on barriers and/or benefits*

Q6. How easy/difficult would it be to use this tool to manage your symptoms?

a. Extremely difficult
b. Somewhat difficult
c. Neutral
d. Somewhat easy
e. Extremely easy

[Interviewer]

- *Ask why they chose their answer*
- *Ask patient and care partner to expand on barriers and/or benefits*

Q7. Is there anything missing that the tool doesn't capture?

[Interviewer]

- *Probe on aspects that they would like to bring to their doctor's attention or record for themselves but is not captured in the tools*

Q8. In the future, what platform(s) would make it easiest to use this tool?

[Interviewer]

- *Probe on whether they would like it to be on a web-based page, a digital app accessible on smartphone and tablet, paper copies, etc*

Q9. What kinds of features would you want to see in this tool?

a. Calendar
b. Alarms
c. Digital diary and note-taking capabilities
d. Voice interaction
e. Other

Part 5: User Interface

Objective: To gather design-specific feedback for future building of the final tool

Q1. What is your general feedback regarding font size, text clarity, colors, and button size?

*[**Interviewer**]*

- *Ask if there is anything they like/dislike, prefer to see, or believe is a missing gap*
- *Probe on their opinion about the number of questions, formatting, layout, errors, visual/sound appeal, pop-outs for medical terminology*

Q2. Are you interested in online or printable questionnaire options?

*[**Interviewer**]*

- *Probe on which option they prefer and why*

Q3. Would you prefer an alternative format?

Q4. Any feedback on the reminder and email functionality aspects of the tool?

Q5. How was navigating through the tool?

*[**Interviewer**]*

- *Probe for their opinion about the order of questions, formatting, layout, errors, visual/sound appeal, pop-outs for medical terminology, self-explanatory nature*

Wrap Up (≈5 minutes)

*[**Interviewer**]*

- *Ask the patient and care partner about final thoughts, questions, and concerns regarding the tool or the interview*

Post-Interview

*[**Interviewer**]*

- *Rate patient's technological proficiency and comfort with technology (or if the care partner provides most assistance, rate the care partner's technological proficiency):*
    - *Requires minimal support*
    - *Requires some support*
    - *Requires substantial support*
- *Comments:* _____

HCP, healthcare professionals.

Supplementary Fig. 1 Digital screenshots of the MY PD-CARE tool prototype.

Welcome and language selection

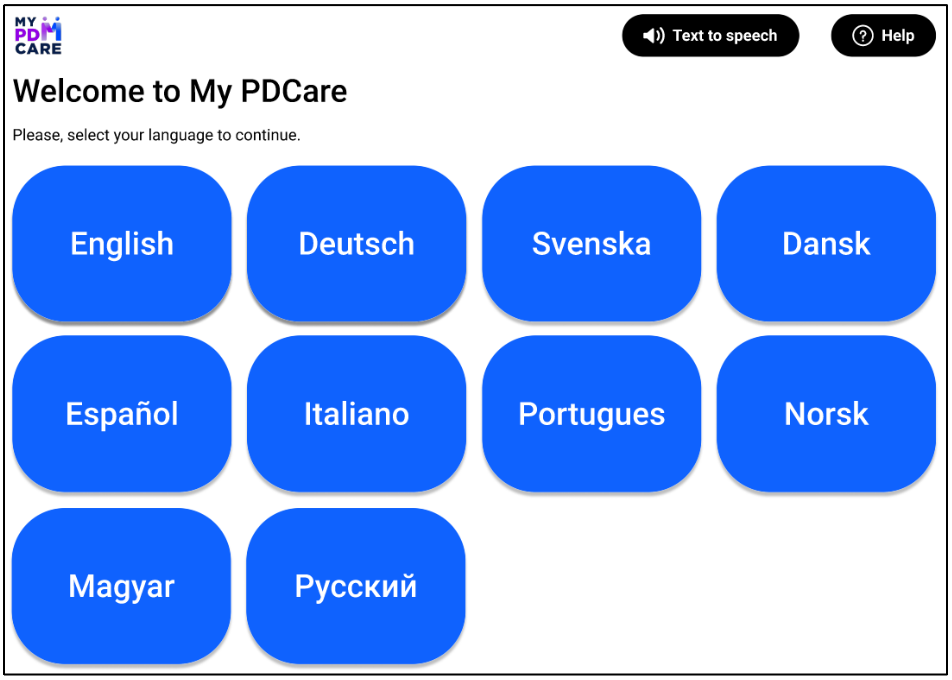

Selection of online or paper-based MY PD-CARE questionnaire

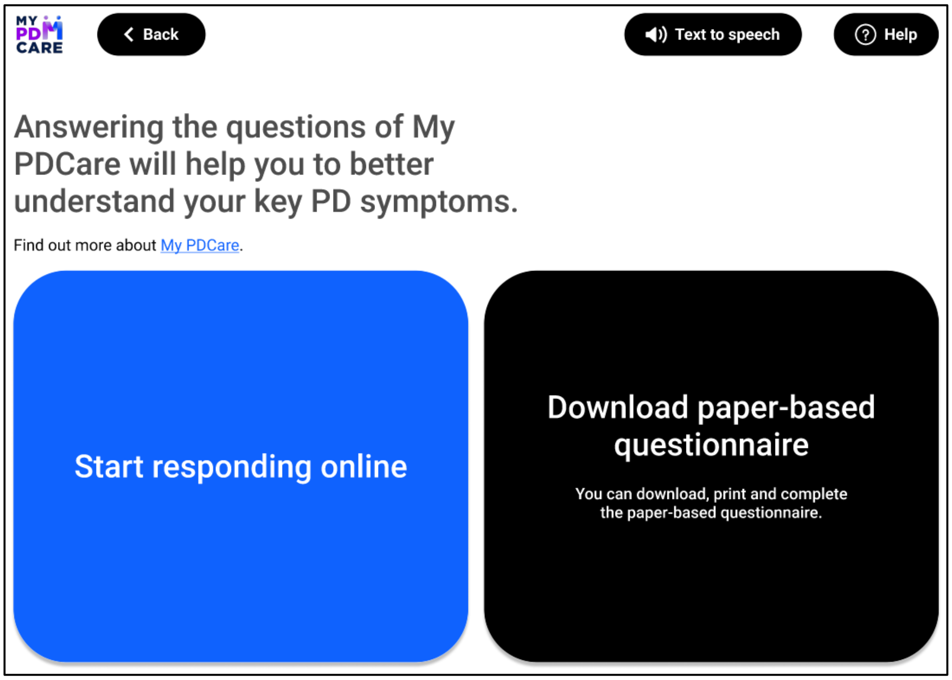

Video Tutorial and Q&A

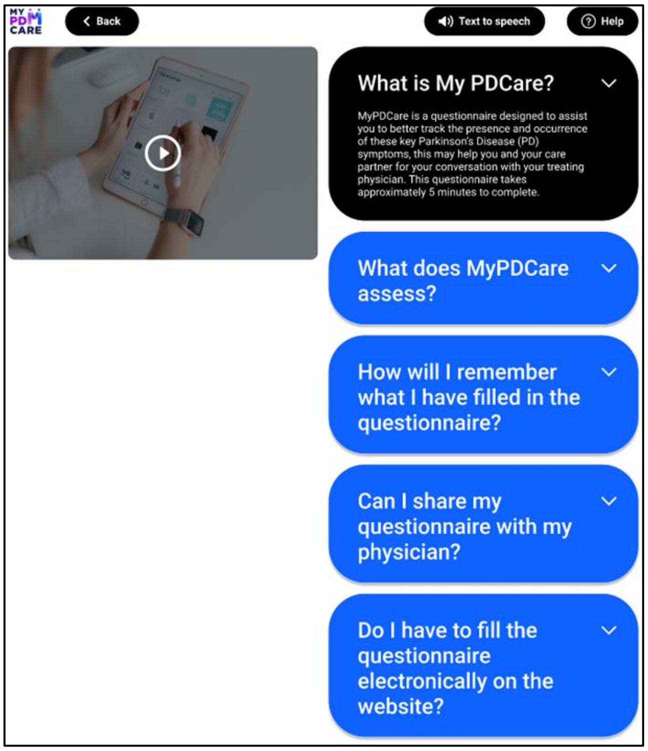

MY PD-CARE ***Question 1***

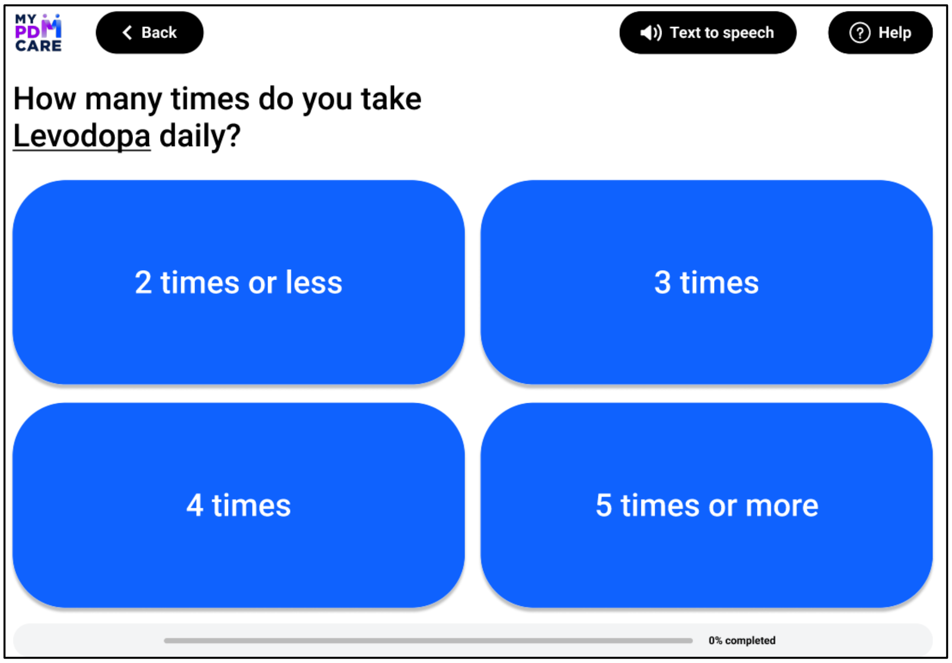

MY PD-CARE ***Question 2***

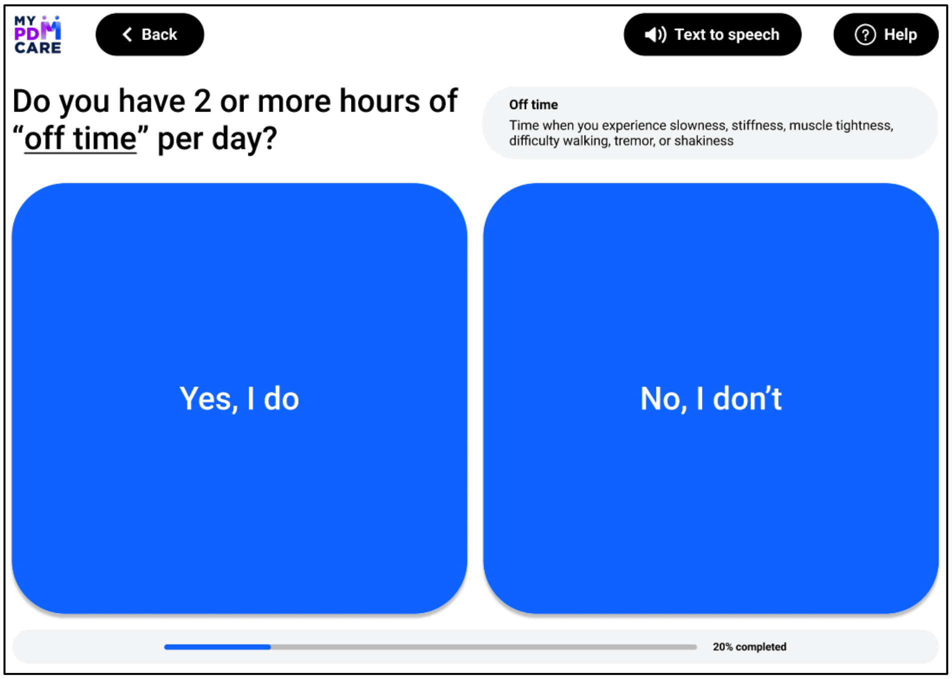

MY PD-CARE ***Question 2a*** *(If the answer to Question 2 was "Yes, I do")*

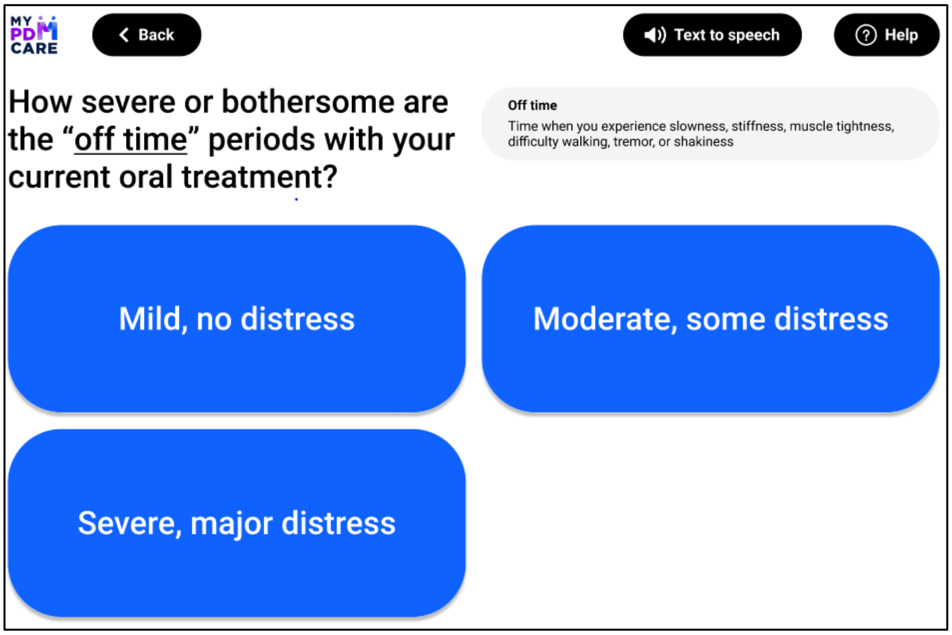

MY PD-CARE ***Question 3***

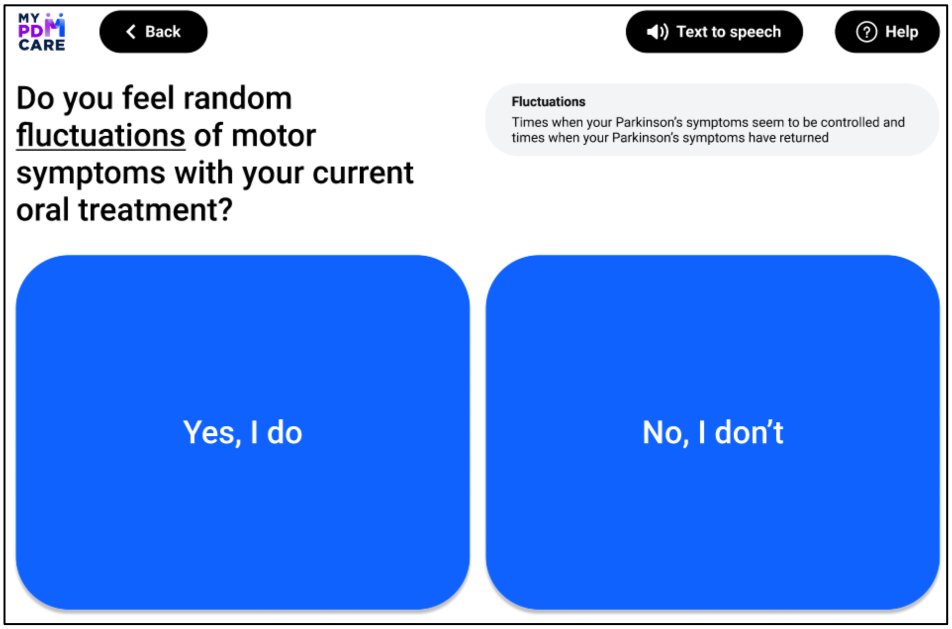

MY PD-CARE ***Question 3a*** *(If the answer to Question 3 was "Yes, I do")*

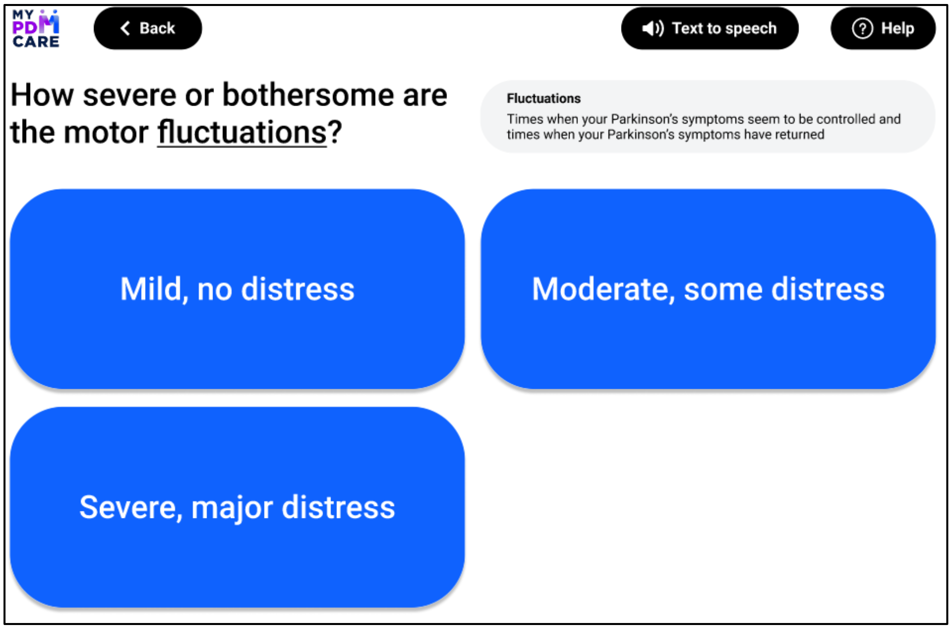

MY PD-CARE ***Question 3b*** *(If the answer to Question 3 was “Yes, I do”)*

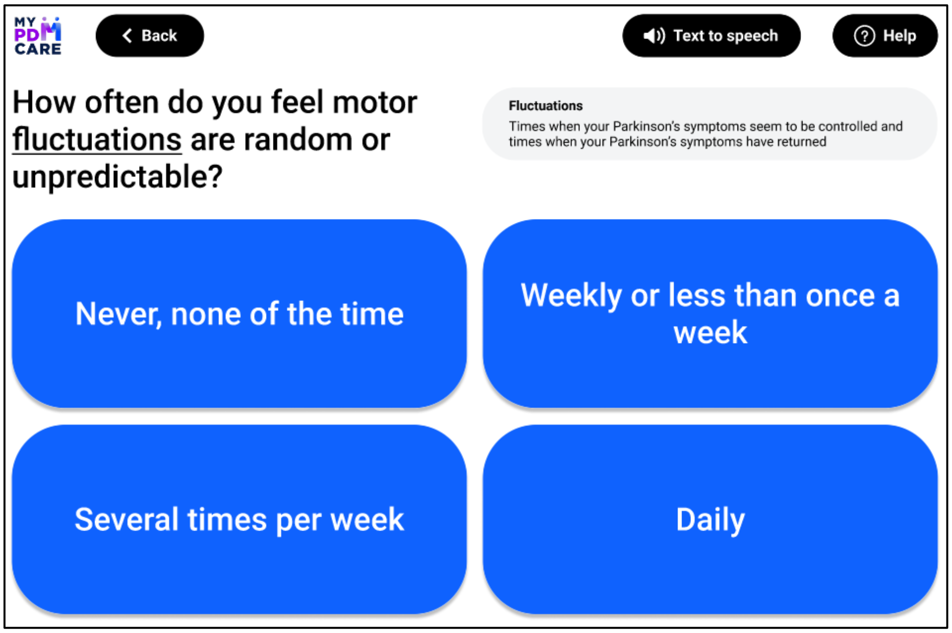

MY PD-CARE ***Question 4***

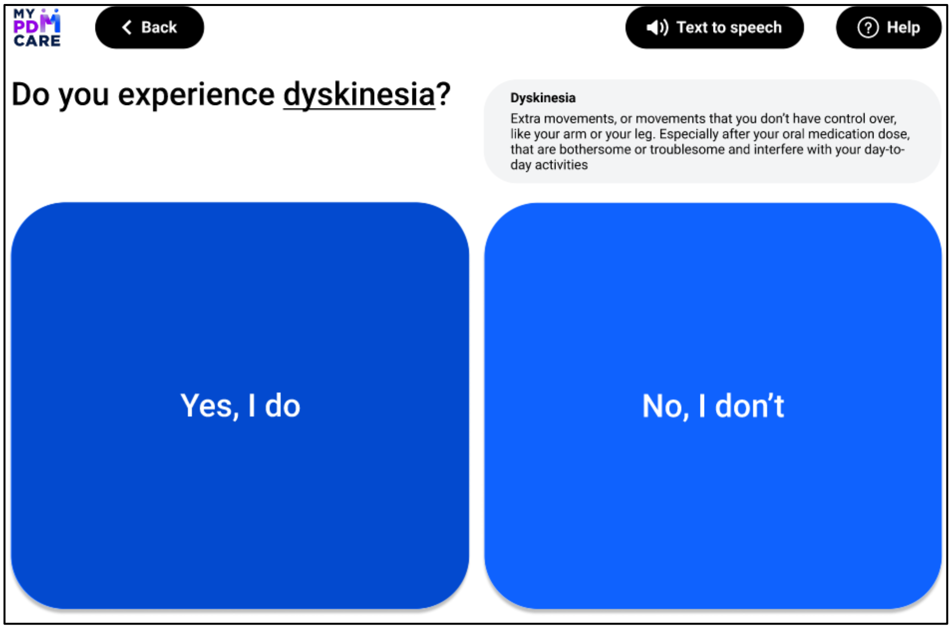

MY PD-CARE ***Question 4a*** *(If the answer to Question 4 was "Yes, I do")*

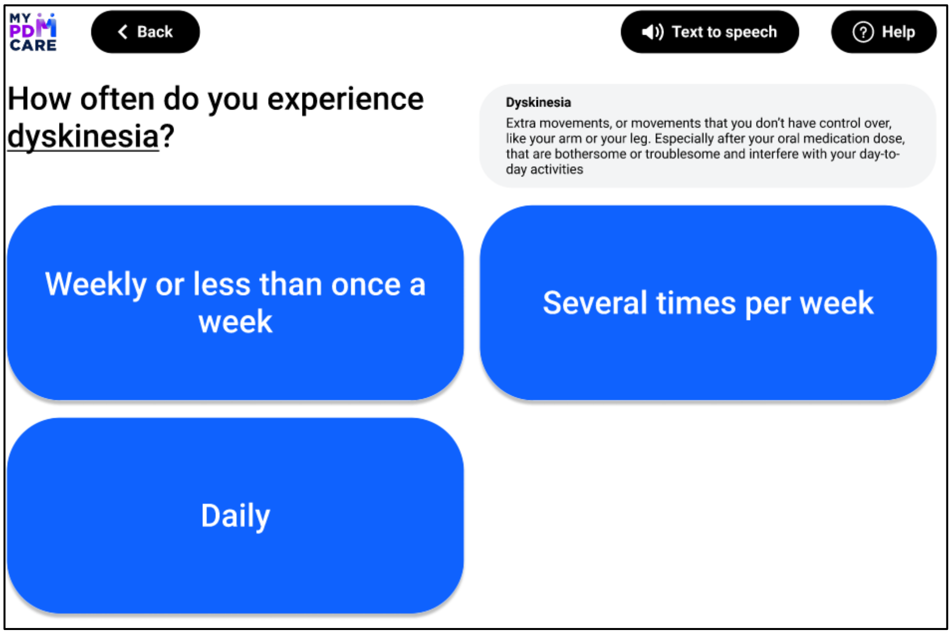

MY PD-CARE ***Question 5***

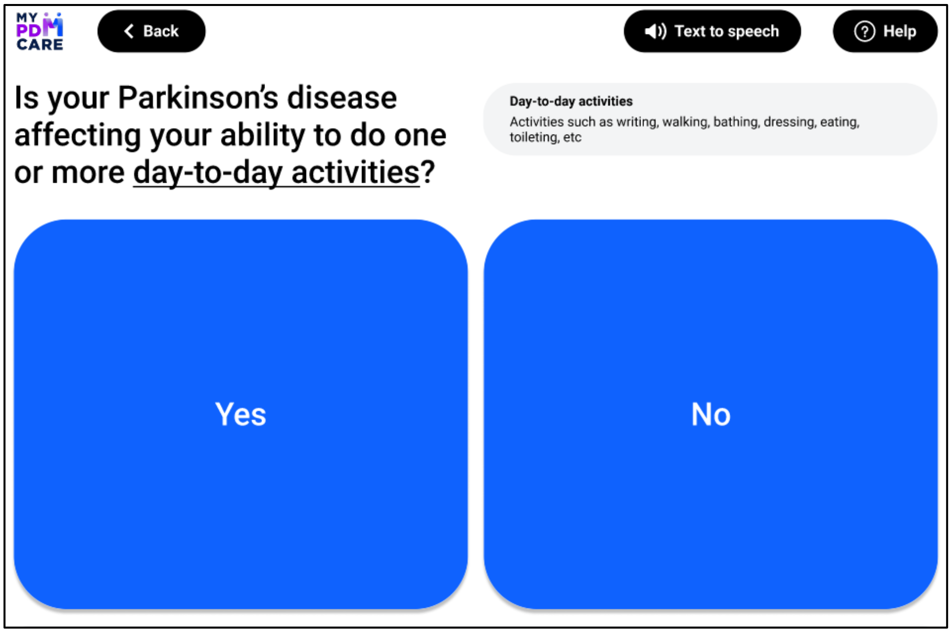

MY PD-CARE ***Question 5a*** *(If the answer to Question 5 was "Yes")*

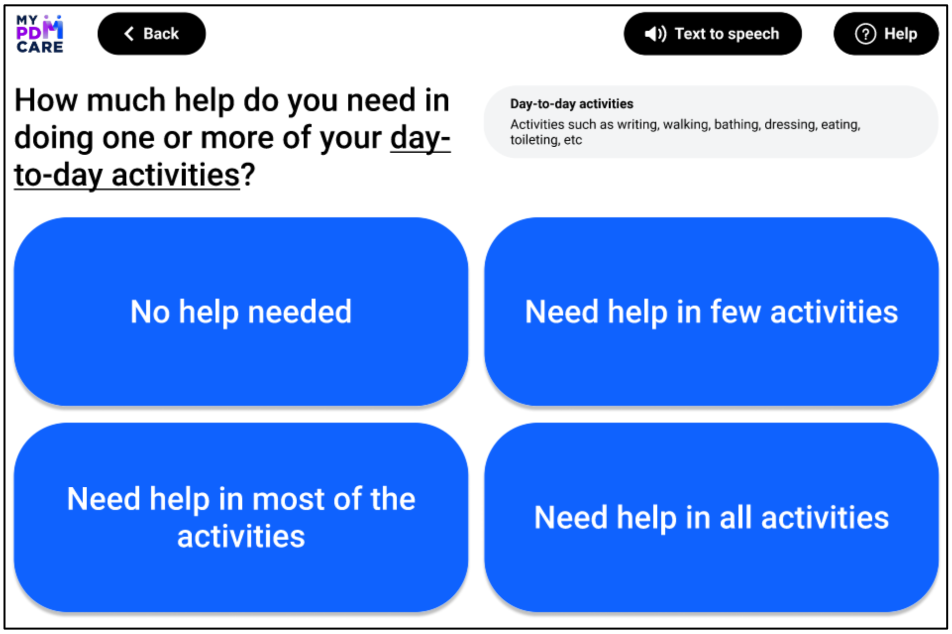

Confirmation of MY PD-CARE questionnaire completion and option to download summary or send summary via email

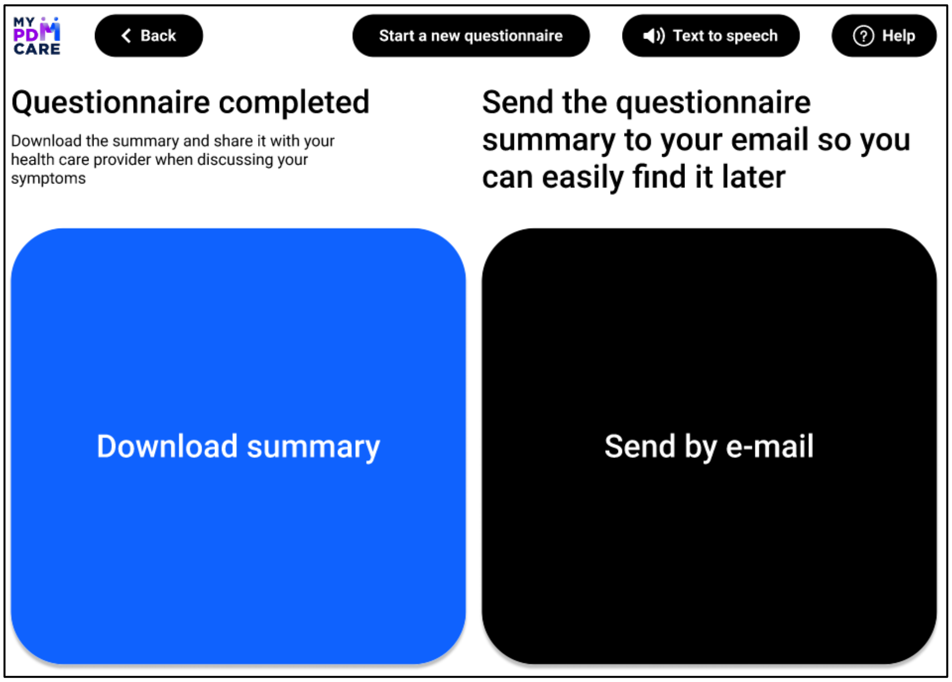

Location to enter email address to receive summary report

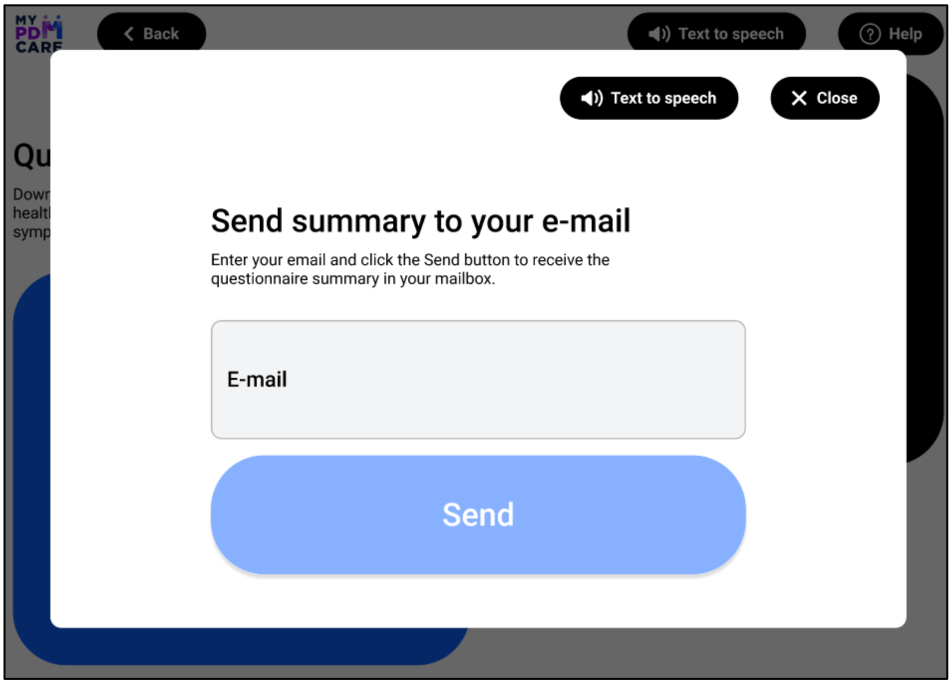

Confirmation that summary report was sent via email and option to set a reminder to use MY PD-CARE tool again before the next appointment

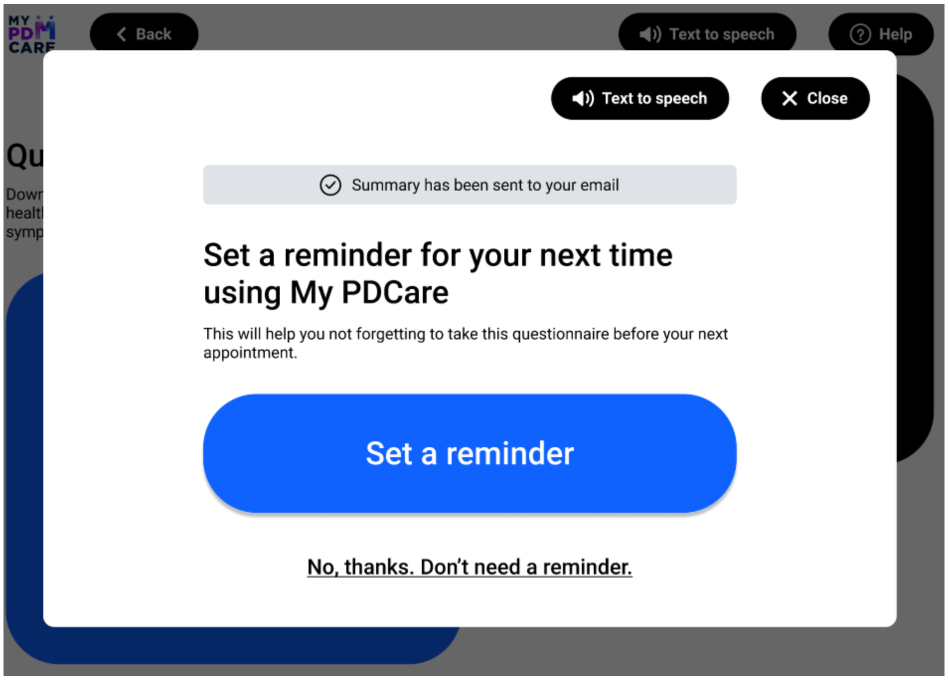

Interface to set the reminder date

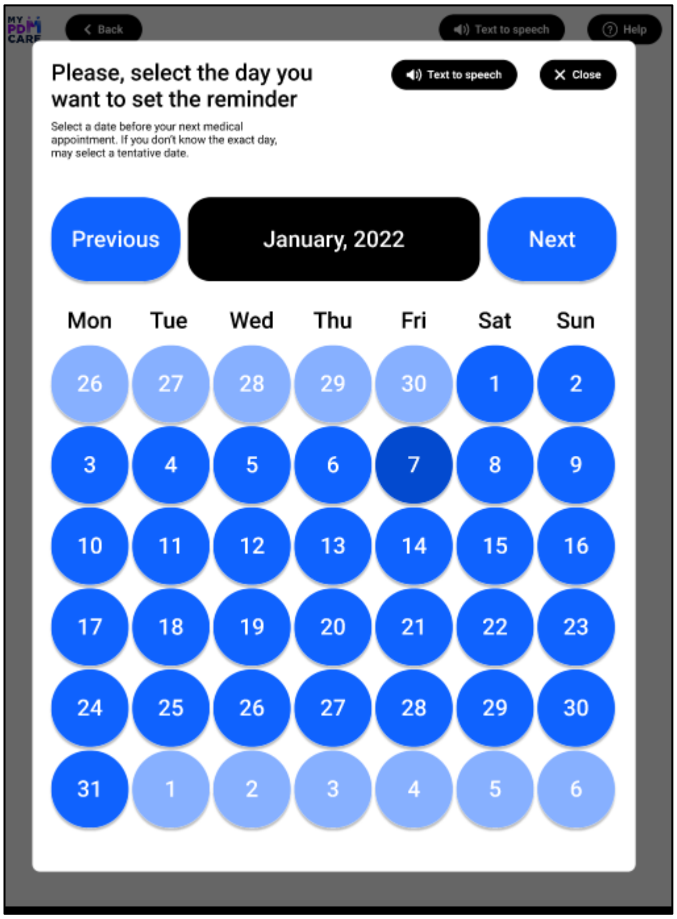

(Example) *confirmation of reminder date*

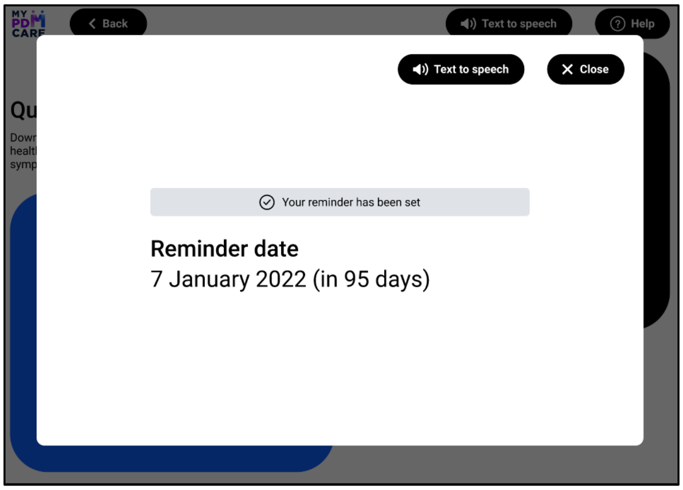

(Example) ***MY PD-CARE summary report*** *(page 1)*

MY PDM CARE

1/2

My PDCare Summary

Name:

Date: 10/10/21

Levodopa dosing frequency	• 3 times per day
2 or more hours of "off time" per day	• Yes<br>• Moderate
Random fluctuations with the current oral treatment	• Yes<br>• Mild<br>• Several times per week
Experiencing Dyskinesia	• Yes<br>• Week, or less than weekly
Day-to-day activities affected	• Yes<br>• Need help in few activities

(Example) ***MY PD-CARE summary report*** *(page 2)*

2/2

Glossary

- **Off time:** Time when you experience slowness, stiffness, muscle tightness, difficulty walking, tremor, or shakiness.
- **Fluctuations:** Times when your Parkinson's symptoms seem to be controlled and times when your Parkinson's symptoms have returned.
- **Dyskinesia:** Extra movements, or movements that you don't have control over, like your arm or your leg. Especially after your oral medication dose, that are bothersome or troublesome and interfere with your day-to-day activities.
- **Day-to-day activities:** Activities such as writing, walking, bathing, dressing, eating, toileting, etc.

Supplementary Fig. 2 Key enhancements of the EU MY PD-CARE digital tool based on SELF-AWARE study participant feedback on the prototype MY PD-CARE digital tool. EU, European Union.

[a]The EU MY PD-CARE Digital Tool is available at: https://www.mypdcare.eu

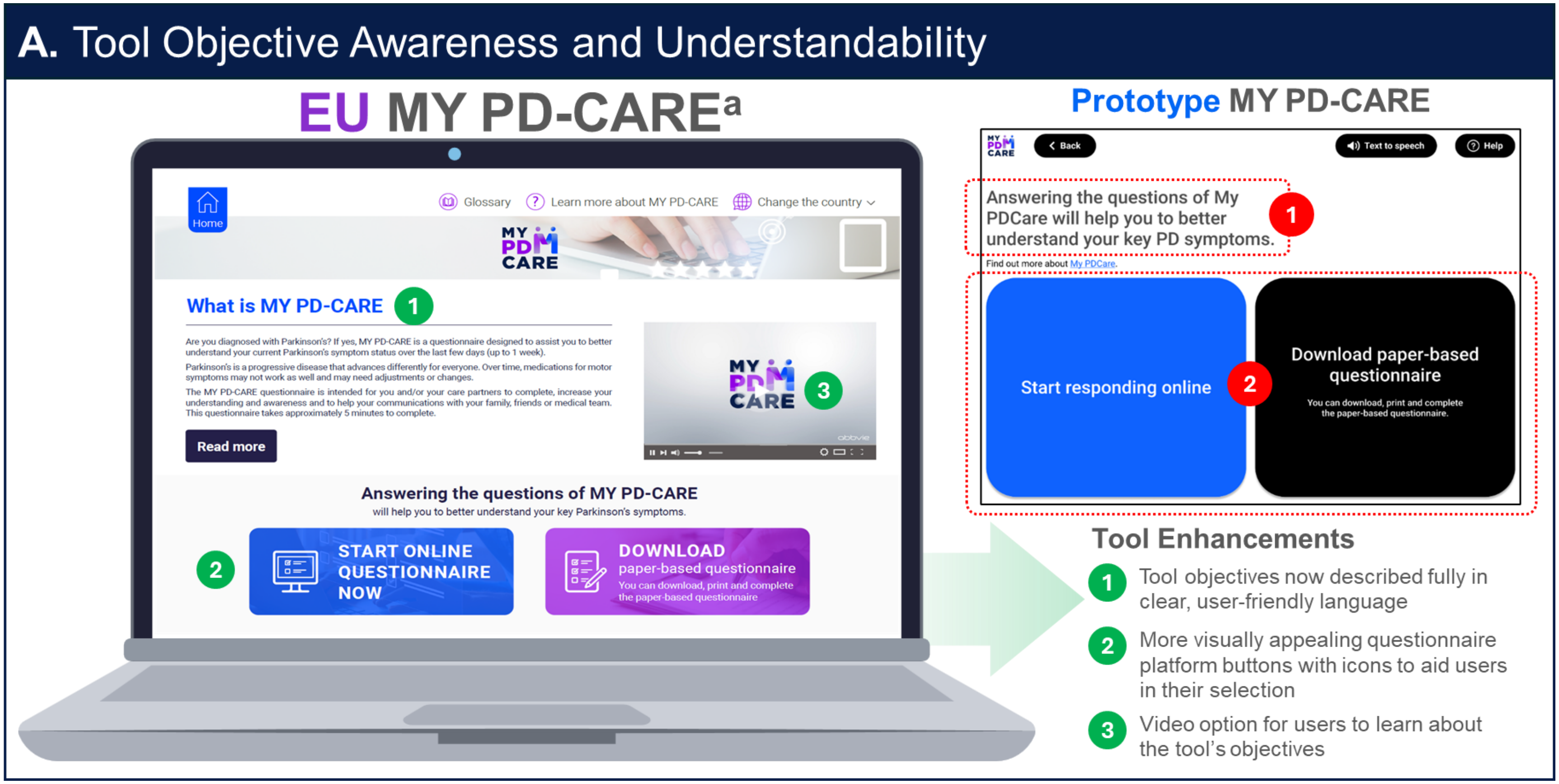

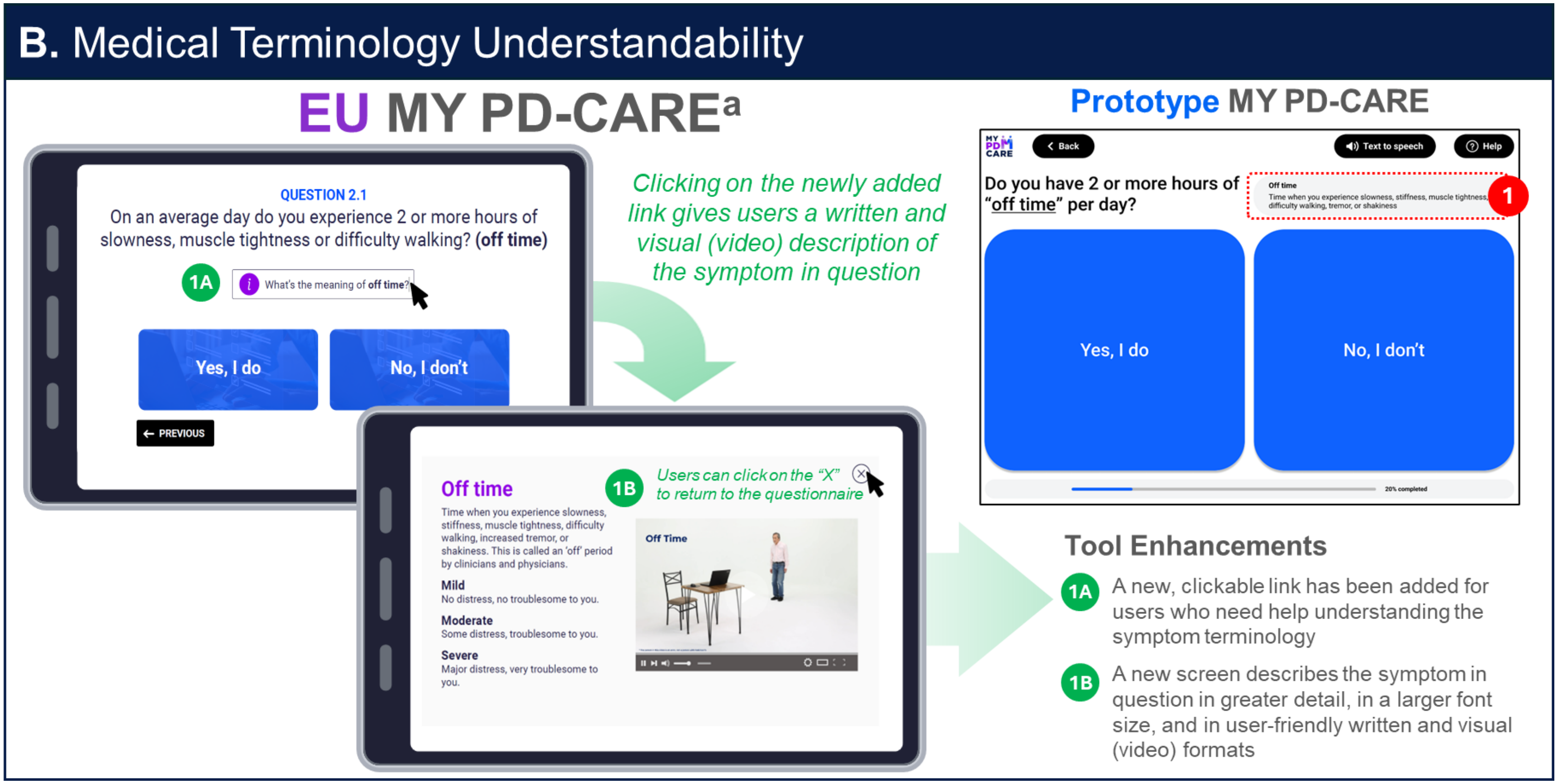
B. Medical Terminology Understandability
EU MY PD-CARE[a]
QUESTION 2.1
On an average day do you experience 2 or more hours of slowness, muscle tightness or difficulty walking? (off time)
1A
What's the meaning of off time?
Yes, I do
No, I don't
PREVIOUS
Clicking on the newly added link gives users a written and visual (video) description of the symptom in question
Off time
1B
Users can click on the "X" to return to the questionnaire
Time when you experience slowness, stiffness, muscle tightness, difficulty walking, increased tremor, or shakiness. This is called an 'off' period by clinicians and physicians.
Mild
No distress, no troublesome to you.
Moderate
Some distress, troublesome to you.
Severe
Major distress, very troublesome to you.
Off Time
Prototype MY PD-CARE
Back
Text to speech
Help
Do you have 2 or more hours of "off time" per day?
Off time
Time when you experience slowness, stiffness, muscle tightness, difficulty walking, tremor, or shakiness
1
Yes, I do
No, I don't
20% completed
Tool Enhancements
1A
A new, clickable link has been added for users who need help understanding the symptom terminology
1B
A new screen describes the symptom in question in greater detail, in a larger font size, and in user-friendly written and visual (video) formats

C. Open-Text Option

EU MY PD-CARE[a]

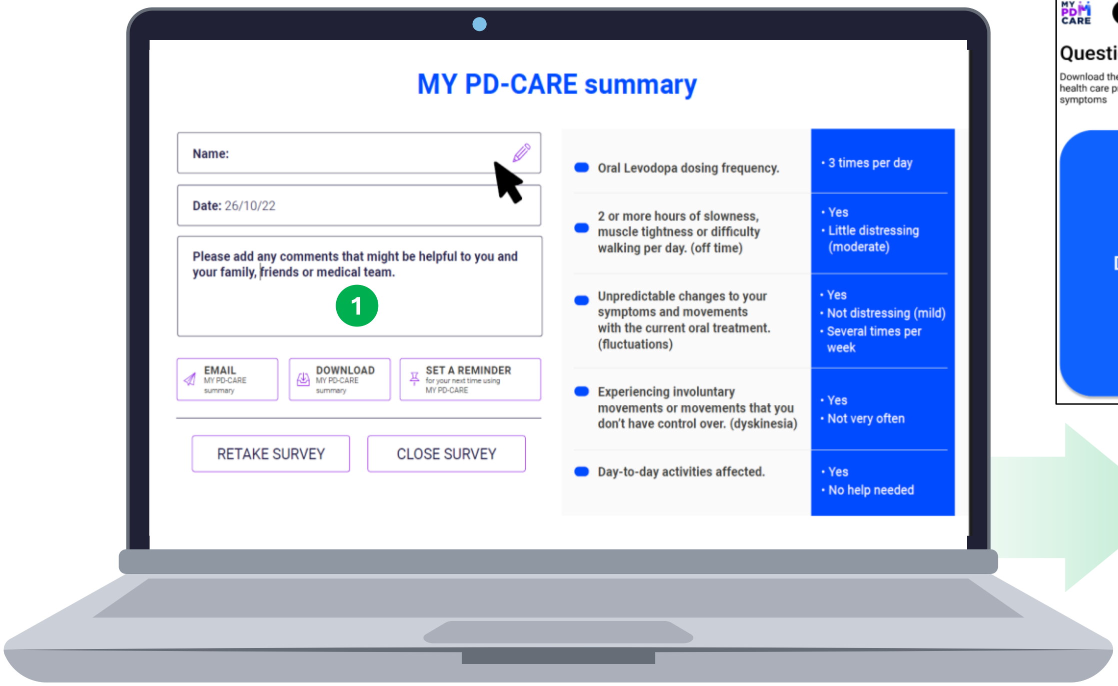

Prototype MY PD-CARE

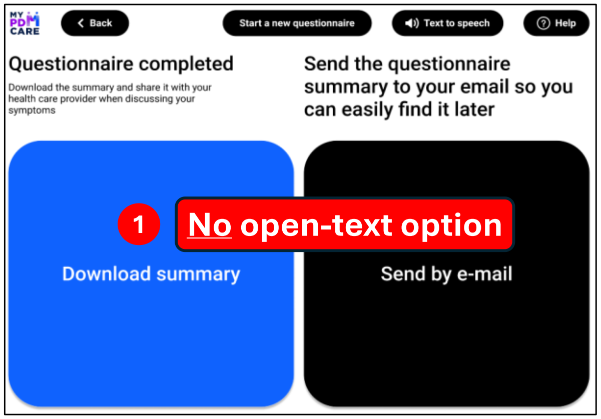

Tool Enhancements

1. New open-text option allows users to include additional info in the MY PD-CARE summary report, such as:
   - Non-motor symptoms (eg, sleep quality)
   - Medication use
   - Reminders to self to ask neurologist at next appointment

Added text is included in the summary report
